# Metabolites Software-Assisted Flavonoid Hunting in Plants Using Ultra-High Performance Liquid Chromatography-Quadrupole-Time of Flight Mass Spectrometry

**DOI:** 10.3390/molecules20033955

**Published:** 2015-03-02

**Authors:** Wan-Yi Gu, Na Li, Elaine Lai-Han Leung, Hua Zhou, Guo-An Luo, Liang Liu, Jian-Lin Wu

**Affiliations:** 1 State Key Laboratory of Quality Research in Chinese Medicine, Macau Institute for Applied Research in Medicine and Health, Macau University of Science and Technology, Avenida Wai Long, Taipa, Macao, China; E-Mails: babyfishhome@163.com (W.-Y.G.); lhleung@must.edu.mo (E.L.-H.L.); hzhou@must.edu.mo (H.Z.); galuo@must.edu.mo (G.-A.L.); lliu@must.edu.mo (L.L.); 2 Faculty of Chinese Medicine, Macau University of Science and Technology, Avenida Wai Long, Taipa, Macao, China

**Keywords:** in-house metabolites software-assisted flavonoid database, biosynthetic pathways, UHPLC-Q-TOF-MS, *Smilax glabra*, flavonoid hunting

## Abstract

Plant secondary metabolism drives the generation of metabolites used for host plant resistance, as biopesticides and botanicals, even for the discovery of new therapeutics for human diseases. Flavonoids are one of the largest and most studied classes of specialized plant metabolites. To quickly identify the potential bioactive flavonoids in herbs, a metabolites software-assisted flavonoid hunting approach was developed, which mainly included three steps: firstly, utilizing commercial metabolite software, a flavonoids database was established based on the biosynthetic pathways; secondly, mass spectral data of components in herbs were acquired by ultra-high performance liquid chromatography-quadrupole-time of flight mass spectrometry (UHPLC-Q-TOF-MS); and finally, the acquired LC-MS data were imported into the database and the compounds in the herbs were automatically identified by comparison of their mass spectra with the theoretical values. As a case study, the flavonoids in *Smilax glabra* were profiled using this approach. As a result, 104 flavonoids including 27 potential new compounds were identified. To our knowledge, this is the first report on profiling the components in the plants utilizing the plant metabolic principles with the assistance of metabolites software. This approach can be extended to the analysis of flavonoids in other plants.

## 1. Introduction

**Figure 1:**
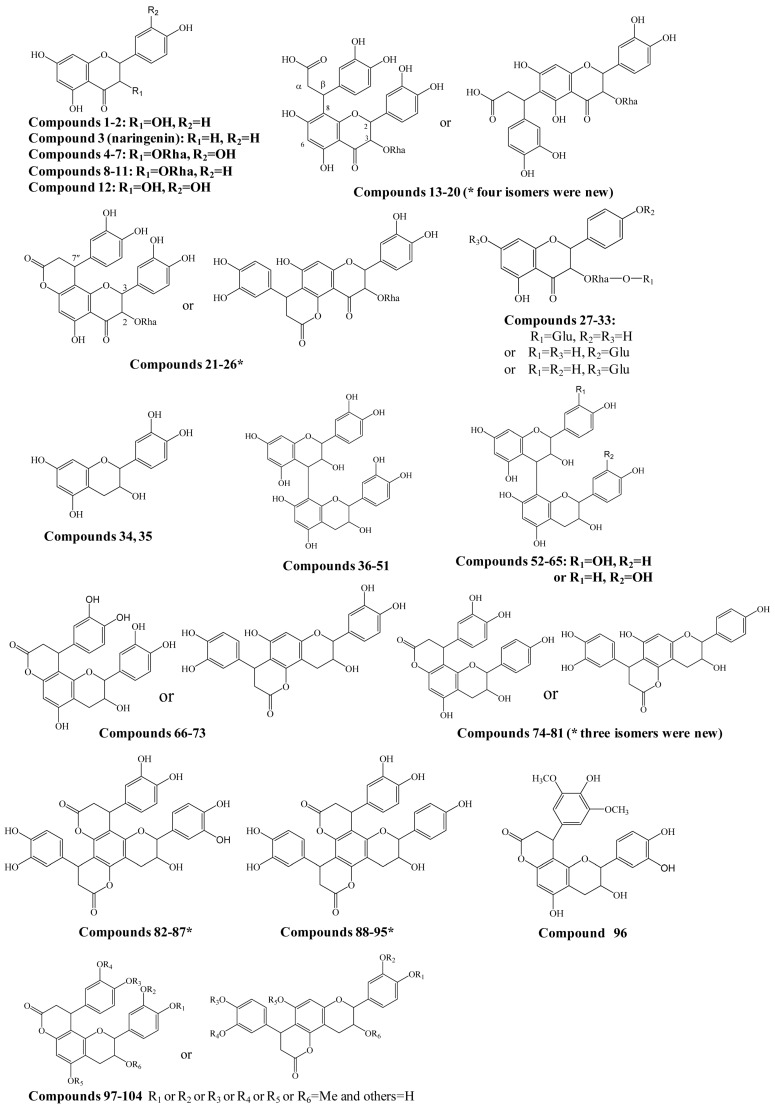
Structures of compounds identified in *Smilax glabra*. * indicated new compounds.

Natural products obtained from herbs or plants are important sources of modern medicines. How to quickly identify the potential bioactive compounds, especially trace components, in herbs is the bottleneck in the study of traditional medicines. Due to the high separation performance and sensitivity and structural identification ability, ultra-high performance liquid chromatography-quadrupole-time of flight mass spectrometry (UHPLC-Q-TOF-MS) had been applied more and more in the study of herbal components. However, the structural determination by the manual and individual interpretation of MS and MS^n^ fragmentation pathways severely depends on the investigator’s knowledge and experience and is a time consuming process. As is commonly known, plants produce secondary metabolites based on specific biosynthetic pathways, which have been widely investigated in the past decades. For example, the biosynthetic pathways of flavonoids had been revealed as starting from the condensation of three molecules of malonyl-CoA and one of 4-coumaroyl-CoA through chalcone synthase and chalcone isomerase to produce a flavanone named naringenin (Figure 1), followed by the production of flavanonol, flavanol, anthocyanidin, successively, by the corresponding specific enzymes (Supplementary Figure S1) [1]. Besides, some functional groups can be further added to the skeletons under the enzymes catalysis, e.g., multihydroxyl-substituted flavanones and flavanonols are produced by the oxidization of hydroxylases (Supplementary Figure S1) [2,3]. The discoveries of flavonoid metabolic principles made it possible to predict the secondary metabolites in the plants. Furthermore, whether the predicted metabolites exist in the plants can be determined by searching their molecular formulae or exact masses using liquid chromatography-mass spectrometry (LC-MS). Using this method, the trace components could be identified too. However, it will still take a long time to manually calculate the molecular formulae or exact masses.

Nowadays, there are several commercial software products for the identification of drug metabolites, e.g., Metabolite ID (Agilent Technologies, Santa Clara, CA, USA) [4], LightSight (Applied Biosystems LLC and MDS Inc., Carlsbad, CA, USA), Profiler-M (Phenomenome Discoveries Inc. Saskatoon, Canada) [5] and CFM-ID web server [6]. Metabolite identification software can not only offer the essential function for automatically identifying and confirming metabolites from data acquired on MS and MS/MS spectra, but also typically contains a built-in editable biotransformation database. For example, Metabolite ID includes 119 biotransformation pathways for phase I and II metabolism reactions. Therefore, using the metabolites software the only step need to set in the whole identification process is to import a structure or formula of a parent compound into the biotransformation database, and then the predicted molecular formula and masses of metabolites are calculated automatically followed by applying the fundamental algorithms of all related mass signals in the acquired MS data. Finally, the predicted metabolites are further confirmed using weighted relevance criteria including isotopic pattern matching, mass defect, extracted compound chromatography (ECC) responses and MS/MS fragment pattern matching. Compared with the traditional manually identification, metabolites software is a simpler, rapid and automatic method for predicting and identifying metabolites. However, until now, the metabolites software has only been used in the study of biosystems, and not applied to the identification of plant secondary metabolites, which have different biotransformation pathways.

Flavonoids are the major class of secondary products produced by higher plants and over 10,000 different members are reported. They show a great diversity of biological and pharmacological activities in human and animals, for example, antioxidant, antitumor, anti-inflammatory and anti-allergic properties [7]. Thus, flavonoids have drawn lots of research interests in “botanicals” for disease therapy and chemoprevention. As mentioned above, the biosynthetic pathways of flavonoids had been well investigated and can be input into the commercial metabolites software packages to establish a database of predicted metabolites. Then, the flavonoids in the plants can be automatically and quickly hunted by the combination of flavonoids database and LC-MS information acquired with the assistance of software. Based on above concept, in this research and as a case study, *Smilax glabra*, a flavonoid-rich traditional medicine, was selected to profile its flavonoids using metabolites software-assisted UHPLC-Q-TOF-MS approach.

## 2. Results and Discussion

### 2.1. Establishment of Flavonoids Database Based on Biosynthetic Principles Using Metabolites Software

As mentioned above, the commercial metabolites software products only contain the biotransformation pathways in animals. Plants have different biosynthetic pathways, so the metabolic principles must be manually input to establish the in-house database.

Most of reported flavonoids in *Smilax glabra* Roxb. belong to two types—the flavanonols and flavanols [8,9,10]. Accordingly, two databases, a flavanonol database and a flavanol database, were established using naringenin and catechin as the parent compounds, respectively. Hydroxylation and glycosylation products were widely detected in this plant, e.g., astilbin, a rhamnoside of 3,5,7,3ʹ,4ʹ-pentadroxyflavanonol (Figure 1), was its major bioactive component [11]. Products of methylation [12,13], prenylation [14,15], butenylation [16], phenylpropanoid-modification [17], and acylation, e.g., galloylation and coumaroylation [18] were usually reported too. Therefore, the following modifiers were considered: hydroxyl, glucosyl and galactosyl [8,9,10], rhamnosyl [8,9,10], apiosyl and arabinosyl [19], and glucuronyl for glycosylation, benzoyl and phenylpropanoyl for acylation, methyl, butyl, prenyl, and phenylpropanoid for alkylation. Modifications by multiple functional groups were also included, e.g., hydroxyl+glucosyl+benzoyl, and the number of modifiers ranged from 1–8.

**Figure 2:**
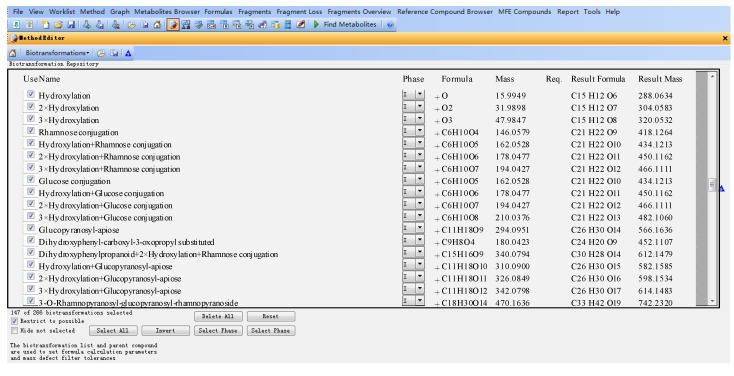
Flavanonol database (parts) established using MassHunter Metabolite ID based on flavonoid metabolic principles.

The databases were established using the MassHunter Metabolite ID software, which mainly includes three parts, the name of the metabolite (Name), the formula and mass of the modifier (Formula and Mass), molecular formula and mass of the assumptive metabolite (Result formula and result mass) (Figure 2).

For example, for eriodictyol or dihydrokaempferol (**1** and **2** in Figure 1), the hydroxylated products from naringenin, the modifier of “+O (oxygen)” was input in “Formula (modifier)”, and then the “Mass (modifier)”, “Result Formula” and “Result Mass” were calculated automatically (Figure 2).

### 2.2. Identification of Flavonoids in Smilax glabra

**Figure 3:**
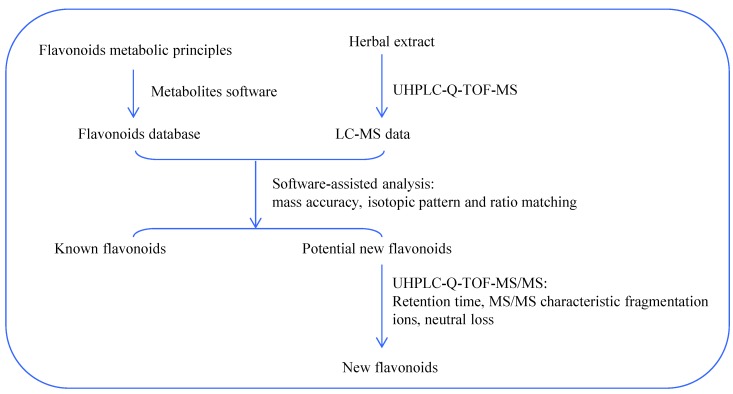
Flow chart of metabolites software-assisted flavonoids hunting in *Smilax glabra*.

In the current study, the flavonoids in *Smilax glabra* were automatically profiled by a combination of in-house flavonoid database as well as UHPLC-Q-TOF-MS analysis. The workflow chart is shown in Figure 3.

**Figure 4:**
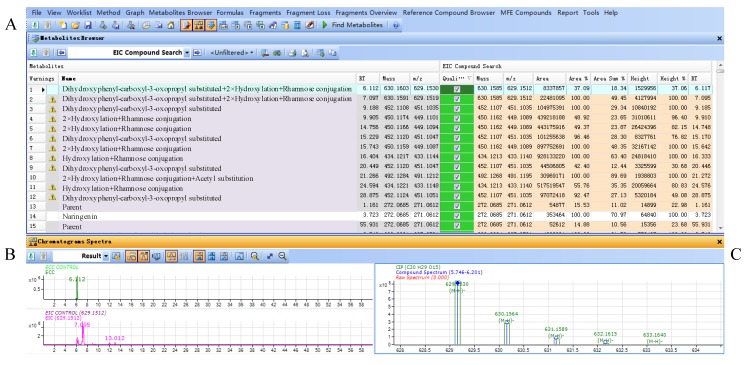
(**A**) Result table for filtered compounds by flavonoid database in *Smilax glabra*; (**B**) ECC and EIC chromatograms of selected compound; (**C**) mass spectrum of selected compound.

Parts of the results obtained from the flavanonols database are shown in Figure 4. The results included three parts, results table (A), extracted compound chromatogram (ECC) and extracted ion chromatogram (EIC) (B) and compounds spectrum (C). The results table (A) displayed all filtered compounds in the database, including the calculated masses, observed masses, abundances, heights and retention times. Part B showed the LC-MS chromatograms for the selected compounds, while the observed mass spectra for the selected compounds were shown in part C.

A total of 454 flavanonols and 170 flavanols were searched, and among them the structures of 104 compounds were further confirmed by the comparison of retention times with suitable standards and/or the interpretation of MS/MS spectra (Figure 1 and Supplementary Table S1). At least 27 potential new compounds were identified as four isomers of dihydroxyphenylpropanoic acid-substituted neoastilbin, astilbin, neoisoastilbin, and isoastilbin (compounds **13**–**20**), dihydroxyphenylpropanoid-substituted astilbin and five isomers (compounds **21**–**26**), three dihydroxyphenylpropanoid-substituted tetrahydroxyflavanol isomers (**74**, **77**, **78**, **80** or **81**), 4,8,10-tris(dihydroxyphenyl)-11-hydroxy-3,4,7,8,11,12-hexahydro-2*H*,6*H*,10*H*-dipyrano[2,3-*f*:2',3'-*h*]chromene-2,6-dione (**82**–**87**), and 4,8-bis(dihydroxyphenyl)-11-hydroxy-10-hydroxyphenyl-3,4,7,8,11,12-hexahydro-2*H*,6*H*,10*H*-dipyrano- [2,3-*f*:2',3'-*h*]chromene-2,6-dione (compounds **88**–**95**) (Figure 1). In addition, by comparison with the corresponding references [8,9,10], engeletin glucoside and isomers (compounds **27**–**33**), 15 isomers of procyanidin B (**36**–**51**), 13 isomers of tetrahydroxyflavan(4→8)-catechin (**52**–**65**), six isomers of cinchonains Ia and Ib (**66**–**73**), three isomers of corbulains Ia and Ib (**75**, **76**, and **79**), and six isomers of smilglabrone B (**97**–**104**) were detected from *Smilax glabra* for the first time (Figure 1 and Supplementary Table S1).

#### 2.2.1. Identification of Flavanonols

Naringenin was selected as parent compound in the flavanonols database. Aside from naringenin, 32 other flavanonols, including at least 10 new structures, were automatically searched by the combination of database and UHPLC-Q-TOF-MS, and further identified by their retention times and MS/MS spectra including characteristic MS/MS fragmentation ions and neutral losses.

*Astilbin, engeletin, and isomers*. Compound **5** at 10.6 min had a quasi-molecular [M-H]^−^ ion at *m*/*z* 449.1107 and was identified as a “2× hydroxylation and rhamnose conjugation” derivative of naringenin, *i.e*., astilbin or an analog. The mass differences between the calculated (theoretical) and observed values were less than 4 ppm for the molecular ion and the other three isotopic ions (Supplementary Table S1) and the abundance ratios of the observed isotopic peaks matched well with the calculated values. Moreover, the retention time was same with that of a standard of astilbin. Thus, compound **5** was definitely identified as astilbin. Similarly, three isomers of astilbin—neoastilbin (**4**), neoisoastilbin (**6**) and isoatilbin (**7**)—as well as engeletin (**9**), were also determined by comparison of retention times and mass spectra with the corresponding standards. The main fragmentation ions in the MS/MS spectra were produced from the neutral losses of a rhamnose substituted at 3-OH (loss of 146 and 164 Da). Besides, compounds **8**, **10** and **11** were deduced as the isomers of engeletin, neoengeletin (**8**), neoisoengeletin (**10**) and isoengeletin (**11**) from the retention times and MS/MS spectra, in which the same fragmentation ions as engeletin were observed [10].

**Figure 5:**
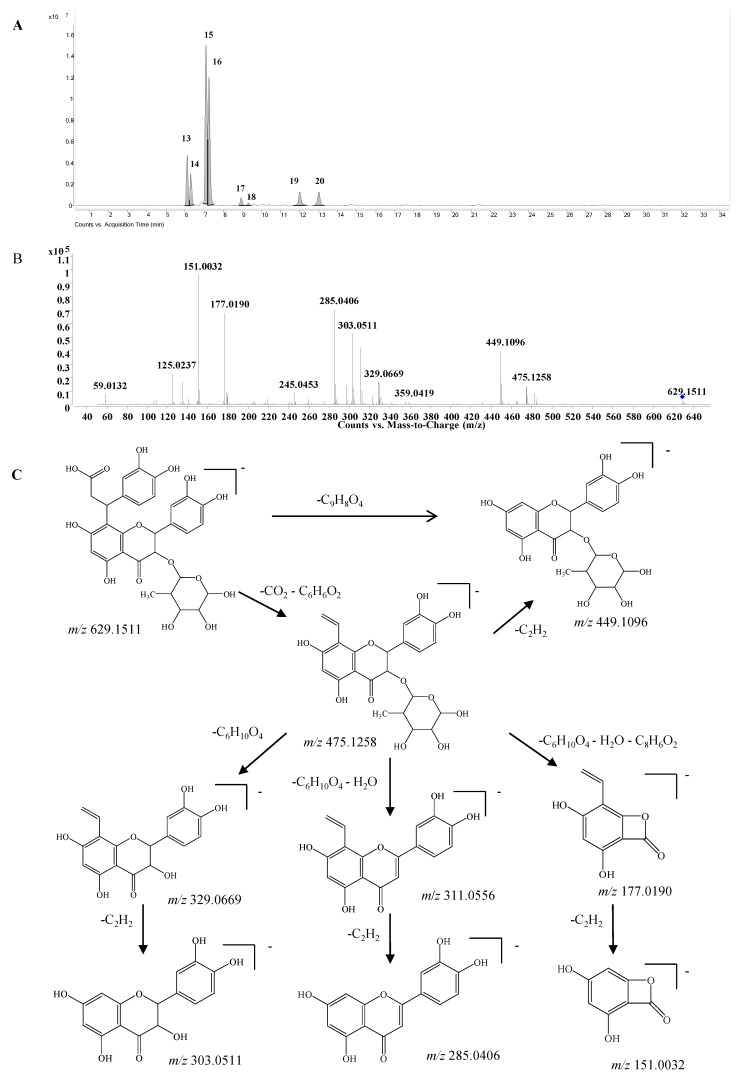
(**A**) Extracted ion chromatogram (EIC) of compound **13**–**20**; (**B**) MS/MS spectrum of compound **13**; (**C**) proposed fragmentation pathway of compound **13**.

*Dihydroxyphenylpropanoic acid-substituted astilbin and isomers*. Eight peaks at 6.1, 6.3, 7.1, 7.2, 8.9, 9.3, 12.0, and 13.0 min (compounds **13**–**20**) with the formula C_30_H_30_O_15_ were identified as the products of “dihydroxylphenylpropanoic acid substituted+2×hydroxylation+rhamnose conjugation” of narigenin. The [M−H]^−^ molecular ion of compound **13** was observed at *m*/*z* 629.1511, along with three isotopic ions at *m*/*z* 630.1541, 631.1554 and 632.1568, which matched well with the calculated values (<5 ppm). Moreover, the abundances of observed ion peaks matched well with the calculated values and the relative errors were 0%–2.5%. The main fragmentation ions at *m*/*z* 303.0511, 285.0406 and 151.0032 indicated that the skeleton should be dihydroquercetin (Figure 5) [20]. The product ions at *m*/*z* 475.1258 and 449.1096 produced from the neutral losses of CO_2_+C_6_H_6_O_2_ and C_9_H_8_O_4_, respectively, confirmed the existence of a dihydroxylphenylpropanoic acid substitution in compound **13**, which connected with the skeleton by C-β. Furthermore, the mass differences of 146 and 164 Da from *m*/*z* 449.1096 to 303.0511 and 285.0406, respectively, demonstrated the presence of a rhamnose unit at 3-OH, which was further confirmed by the product ions at *m*/*z* 329.0669 and 311.0535 produced from *m*/*z* 475.1258. The product ion at *m*/*z* 177.0190 indicated that the dihydroxylphenylpropanoic acid group should be substituted at C-6 or C-8 (Figure 5C). Therefore, compound **13** was identified as β-dihydroxyphenyl-α-carboxyl-3-oxopropyl-substituted 3,5,7,3ʹ,4ʹ-pentahydroxyl-flavanonol 3-*O*-rhamnoside, *i.e*., dihydroxyphenylpropanoic acid-substituted astilbin or an isomer. Compounds **15** and **16** had similar MS and MS/MS spectra as compound **13** (Supplementary Table S1), and were identified as isomers of compound **13**, where the differences might be the configurations at C-2, C-3 and C-β, or the substitution position of the dihydroxyphenylpropanoic acid moiety on ring A. In addition, five trace compounds, **14**, and **17**–**20** were also assigned as the same structure by the software-assisted mass data matching.

These eight compounds can be divided into four groups from retention times 6.1 min (**13**) and 6.3 min (**14**); 7.1 min (**15**) and 7.2 min (**16**); 8.9 min (**17**) and 9.3 min (**18**); 12.0 min (**19**) and 13.0 min (**20**), and the retention times within the groups were very close, while they were significantly different among the groups. The pattern was very similar to that of astilibin and three isomers, in which the elution order was neoastilbin (9.9 min), astilbin (10.6 min), neoisoastilbin (14.8 min) and isoastilbin (15.9 min). Thus, **13** and **14** should be β-dihydroxyphenyl-α-carboxyl-3-oxopropyl-substituted neoastilbin, and the difference lies on the configuration of C-β or the substitution position of the dihydroxyphenylpropanoic acid unit on ring A. Similarly, six other compounds were identified as β-dihydroxyphenyl-α-carboxyl-3-oxopropyl-substituted astilbins (**15** and **16**), β-dihydroxyphenyl-α-carboxyl-3-oxopropyl-substituted neoisoastilbins (**17** and **18**), and β-dihydroxyphenyl-α-carboxyl-3-oxopropyl-substituted neoisoastilbins (**19** and **20**). Four of them were detected for the first time [10].

**Figure 6:**
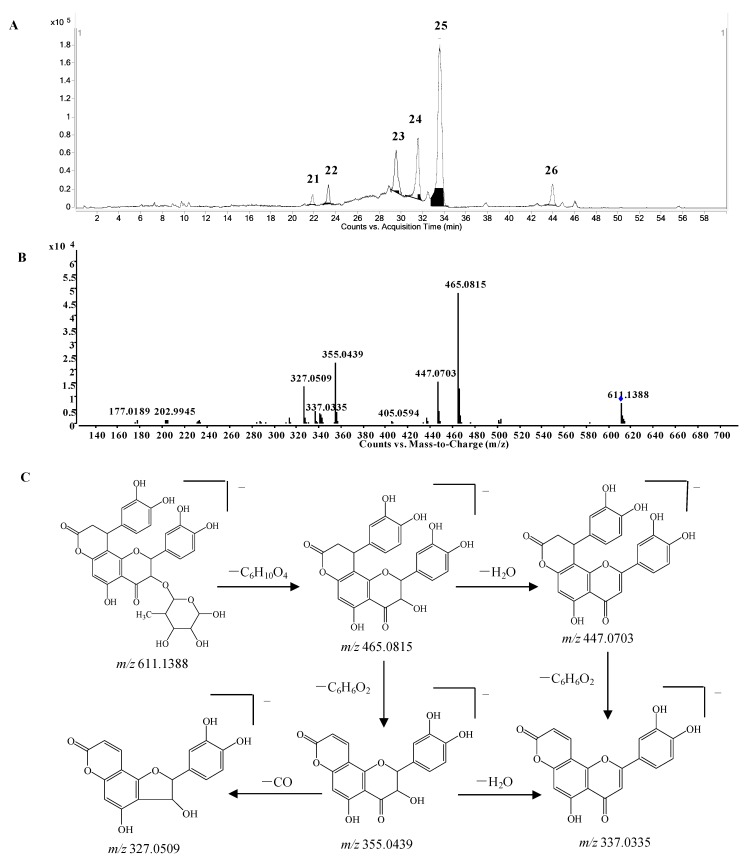
(**A**) Extracted ion chromatogram (EIC) of compounds **21**–**26**; (**B**) MS/MS spectrum of compound **24**; (**C**) proposed fragmentation pathway of compound **24**.

*Phenylpropanoid-substituted astilbin and isomers.* Six peaks at 21.9, 23.3, 29.5, 31.5, 33.7 and 43.8 min (compounds **21**–**26**) with the molecular formula of C_30_H_28_O_14_ were identified as the products of “dihydroxyphenylpropanoid-substituted+2×hydroxylation+rhamnose conjugation” of narigenin. The [M−H]^−^ molecular ion of compound **24** was observed at *m*/*z* 611.1388, and three isotopic ions at *m*/*z* 612.1419, 613.1434 and 614.1438, which matched well with the calculated values, were also seen. Moreover, the abundances of the observed ion peaks matched well with the calculated values and the relative errors were 0%–4.5%. The main fragmentation ions at *m*/*z* 465.0815 [M-H-rhamnose]^−^ and 447.0703 [M-H-rhamnose-H_2_O]^−^ (Figure 6B) confirmed the presence of rhamnose, while the ions at *m*/*z* 355.0439 [M-H-rhamnose-C_6_H_6_O_2_]^−^ and 337.0335 [M-H-rhamnose-H_2_O-C_6_H_6_O_2_]^−^ indicated the substitution of the dihydroxyphenyl group. The molecular formula C_30_H_28_O_14_ has two hydrogens less and one oxygen more than that of glabraoside A (C_30_H_30_O_13_), a dihydroxyphenylpropanoid-substituted catechin rhamnoside, and the fragmentation ions produced from the losses of rhamnose and dihydroxyphenyl group were also 14 Da more than the corresponding ions in glabraoside A [19], so it should be a dihydroxyphenylpropanoid-substituted flavanonol rhamnoside, *i.e*., dihydroxyphenyl-propanoid-substituted astilbin or an isomer. The product ions at *m/z* 327.0509 produced from the loss of CO from the ion of *m/*z 355.0439 further confirmed the existence of a carbonyl group at position 4 in compound **24**. Compounds **23**, **25** and **26** were identified as isomers of compound **24** by their similar MS and MS/MS spectra (Supplementary Table S1), and the differences among them should be the configurations at C-2, C-3 and C-7", or the substitution position of the dihydroxyphenylpropanoid unit on ring A. This is the first report of phenylpropanoid-substituted astilbin and isomers.

*Engeletin glucoside and isomers.* Compounds **27**–**33** with the molecular formulae C_27_H_32_O_1__5_ were identified as “hydroxylation+rhamnose+glucose conjugation” derivatives of narigenin. The exact masses of the molecular ions matched well with the theoretical values with an error of less than 5 ppm, and at least two isotopic peaks were observed, and their exact masses and abundances were consistent with the calculated values too (Supplementary Table S1). These compounds have one glucose more than engeletin and isomers, neoengeletin, neoisoengeletin, and neoisoengeletin. According to the retention times, they were divided into four groups, 11.7 min (**27**) and 11.9 min (**28**); 12.3 min (**29**) and 12.7 min (**30**); 14.6 min (**31**) and 15.0 min (**32**); and 16.9 min (**33**).

The pattern of these groups was very similar to that of neoengeletin (16.6 min), engeletin (19.2 min), neoisoengeletin (20.9) and isoengeletin (24.6 min), moreover, the retention times were shorter than those of the corresponding engeletin and isomers, so it was deduced that they were neoengeletin glucoside (**27** and **28**), engeletin glucoside (**29** and **30**), neoisoengeletin glucoside (**31** and **32**) and isoengeletin glucoside (**33**). The glucose might be a substituent at 7-OH, 4ʹ-OH or the rhamnose. No compound with the molecular formula of C_27_H_32_O_1__5_ was previously reported from *S. glabra*.

#### 2.2.2. Identification of Flavanols

The flavanol database was established using catechin as parent compound. Two peaks (**34** and **35**) with the molecular ions at *m/z* 289.0718 (C_15_H_13_O_6_) were detected at 3.8 and 4.8 min. They had identical fragmentation ions at *m/z* 245.08, 205.05, and 179.03, which were same as those of catechin [8]. Epicatechin eluted later than catechin under similar liquid chromatography conditions [8], so compounds **34** and **35** were identified as catechin and epicatechin, respectively.

*Flavanol polymers*. Compounds **36**–**51** with the molecular formula of C_30_H_26_O_12_ (*m/z* 577.13) were identified as “catechin conjugation” products of catechin with the assistance of the software by comparison of MS data (Table S1). The MS/MS spectra further confirmed the structures, in which the main fragmentation ions were observed at *m/z* 289.07, 407.07, 425.08, and 451.10 similar to those of procyanidin B [8,10]. Thus, compounds **36**–**51** were identified as dimers of catechin, *i.e*., procyanidin B and isomers. Similarly, compounds **52**–**65** with the molecular formula of C_30_H_26_O_11_ (*m/z* 561.1402) were deduced to be “tetrahydroxyflavanol conjugation” derivatives of catechin and isomers. They had one oxygen less than compounds **36**–**51** (Supplementary Table S1). Unlike procyanidin B, the characteristic fragmentation ion of a catechin dimer at *m/z* 451.10 was not observed [8,10], and instead an ion at *m*/*z* 435.10 was observed in the MS/MS spectra of compounds **52**–**54**, thus the upper unit of these three polymers should be tetrahydroxyflavanol, while the lower unit was catechin. This was further confirmed by the fragmentation ion at *m*/*z* 407.07 produced from the loss of ring-B-^2^CH=^3^CH-OH of the upper unit (C_8_H_10_O_3_) [21]. Therefore, compounds **52**–**54** were identified as tetrahydroxyflavanol(4→8)-catechin and isomers. Compounds **55**–**65** should be considered polymers of catechin and tetrahydroxyflavanol, although the linkage couldn’t be determined due to the lack of MS/MS information. Until now, just one isomer of C_30_H_26_O_12_ (procyanidin B) and one of C_30_H_26_O_11_ were found in *S. glabra* [8,10], accordingly, the other 28 isomers were identified in this herb for the first time in this work.

*Dihydroxyphenylpropanoid-substituted catechin and isomers.* Compounds **66**–**73** with the molecular formula of C_24_H_20_O_9_ (*m/z* 451.1035) at 9.2, 15.2, 15.4, 15.8, 20.5, 20.9, 28.9, and 30.4 min were assigned as “dihydroxyphenylpropanoid conjugation” products of catechin. They had similar MS/MS spectra, and the main product ions were observed at *m/z* 341.06 and 217.01, which were similar to those of cinchonain Ib [8,10]. The ion at *m/z* 341.06 was produced from the neutral loss of C_6_H_6_O_2_ (110 Da), which confirmed the existence of a dihydroxyphenyl group. The ion at *m/z* 217.01 was generated from the neutral losses of C_6_H_6_O_2_ and C_7_H_8_O_2_ (234 Da), which was produced from the elimination of dihydroxytoluene [19]. Therefore, compounds **66**–**73** were confirmed to be dihydroxylphenylpropanoid-substituted catechins and isomers, *i.e*., cinchonains Ia, Ib, Ic, Id and isomers (Supplementary Table S1). Until now, only two isomers, cinchonains Ia and Ib, were reported from *S. glabra* [8,10].

*Dihydroxyphenylpropanoid-substituted tetrahydroxyflavnol and isomers*. Compounds **74**–**81** with the molecular ions of *m*/*z* 435.10 at 13.7, 17.3, 21.9, 22.5, 23.2, 24.1, 39.6, and 40.2 min were assigned as “phenylpropanoid+hydroxylation” derivatives of catechin. The molecular formula C_24_H_20_O_8_ has one oxygen less than compounds **66**–**73** (Supplementary Table S1). Like cinchonain Ib, the main fragmentation ion of compounds **74**, **77**, **78**, **80** and **81** at *m/z* 325.0730 was produced from the neutral loss of C_6_H_6_O_2_. It was reported that the ion produced from the neutral loss of benzene or substituted benzene was the characteristic product ion of phenylpropanoid-substituted catechins, e.g., the neutral loss of C_6_H_6_O_2_ (110 Da) was the characteristic fragmentation ion of a 3,4-dihydroxyphenylpropanoid-substituted catechin. Thus, the dihydroxyphenyl group should be a substituent at C-7ʺ, not C-2, *i.e*., a dihydroxyphenylpropanoid-substituted tetrahydroxyflavanol. Until now, there were only two such isomers, corbulain Ia and Ib, that were reported from *S. corbularia* [22]. Three compounds among **74**, **77**, **78**, **80** and **81** were potential new compounds, and the differences among them should be the configurations of C-2, C-3, and C-7ʺ, and the substitution position of the dihydroxyphenylpropanoid moiety on ring A.

*Bis-dihydroxyphenylpropanoid-substituted catechin and isomers.* Compounds **82**–**87** had the same molecular formula of C_33_H_26_O_12_, and were identified as “2×dihydroxyphenylpropanoid-substituted” products of catechin. The major fragmentation ions in the MS/MS spectra were observed at *m/z* 503.10, 393.06, and 341.06 (Supplementary Table S1). The product ions at *m/z* 503.10 and 393.06 were attributed to the neutral losses of one and two molecules of C_6_H_6_O_2_ (110 Da), respectively, which indicated the existence of two dihydroxylphenyl groups. Another major product ion at *m/z* 341.06 produced from the neutral loss of C_6_H_6_O_2_+C_9_H_6_O_3_ was also observed as prominent fragmentation peak in MS/MS spectrum of cinchonain Ib. Hence, compounds **82**–**87** should be bis-dihydroxylphenylpropanoid-substituted catechins, which were further confirmed by the fragmentation ions at *m/z* 451.1037 (C_24_H_19_O_9_) produced from the neutral loss of C_9_H_6_O_3_. The structure of one compound was determined by Liquid Chromatography-Solid Phase Extraction-Nuclear Magnetic Resonance (LC-SPE-NMR) approach in our laboratory.

**Figure 7:**
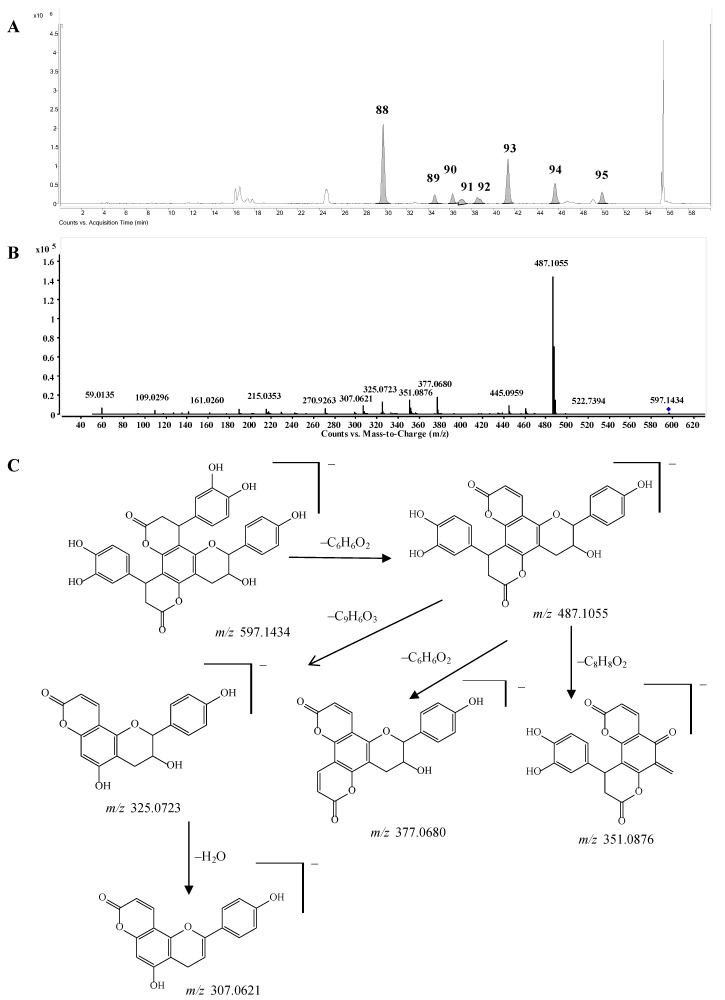
(**A**) Extracted ion chromatogram (EIC) of compounds **88**–**95**; (**B**) MS/MS spectrum of compound **88**; (**C**) Proposed fragmentation pathway of compound **88**.

*Bis-dihydroxyphenylpropanoid-substituted tetrahydroxyflavanol and isomers.* Compounds **88**–**95** had the same molecular formula C_33_H_26_O_1__1_, and were predicted as “2×phenylpropanoid-substituted+3×hydroxylation” products of catechin. Like compounds **82**–**87**, the main fragmentation ions of compound **88** were produced from the neutral losses of one and two C_6_H_6_O_2_ and C_6_H_6_O_2_+C_9_H_6_O_3_ at *m/z* 487.1055, 377.0680, and 325.0723, respectively, which indicated the substitution of two dihydroxyphenylpropanoid units (Figure 7C). The fragmentation ion at *m*/*z* 351.0876 produced from the loss of ring-B-^2^CH=^3^CH-OH from the ion of *m*/*z* 487.1055 further showed that only one hydroxyl group was substituted on ring B of compound **88**, so compound **88** was determined as a bis-dihydroxyphenylpropanoid-substituted tetrahydroxyflavanol, *i.e*., 4,8-bis(dihydroxy-phenyl)-11-hydroxy-10-hydroxyphenyl-3,4,7,8,11,12-hexahydro-2*H*,6*H*,10*H*-dipyrano[2,3-*f*:2',3'-*h*]-chromene-2,6-dione. The other compounds **89**–**95** might be the isomers of compound **88** from the MS data (Supplementary Table S1), however, the substitution positions of the hydroxyl group couldn’t be determined due to the lack of MS/MS spectra. This is the first report concerning this type of structures.

*Methylated cinchonains*. Eight peaks at 24.3, 25.3, 25.5, 25.9, 27.2, 31.4, 32 and 38.5 min (compounds **97**–**104**) had the same molecular formula of C_25_H_22_O_9_ and were indicated as “methylation+dihydroxyphenylpropanoid-substituted” derivatives of catechin (Supplementary Table S1). The MS/MS spectrum of compound **99** was almost same with that of cinchonain Ib, while the molecular formula had one methylene more than cinchonain Ib. Due to the characteristic fragmentation ions related to the loss of benzene from a dihydroxyphenylpropanoid unit, the methyl group should thus be on 3ʺ-OH or 4ʺ-OH, *i.e*., smiglabrone B or an isomer (Figure 1) [9]. The difference among the other isomers (compounds **97**–**98** and **100**–**104**) should be the substitution position of the methyl group or the configuration of the cinchonain skeletons.

Until now, only one such compound, smiglabrone B, was reported in *S. glabra* [9] and a stereoisomer of smiglabrone B was isolated from *Guibourtia coleosperma* [23]. Besides, a compound **96** with the molecular formula of C_26_H_24_O_10_ was assigned as a “2×methylation+hydroxylation+ dihydroxyphenylpropanoid-substituted” derivative of catechin, *i.e*., smiglabrone A or an isomer (Figure 1) [9].

## 3. Experimental Section

### 3.1. Chemicals and Reagents

Astilbin, isoastilbin, neoastilbin, neoisoastilbin, and engeletin were purchased from Chengdu Push Bio-Technology Co., Ltd (Chengdu, China), and the purities were higher than 95%. MS grade acetonitrile, methanol and water were purchased from J.T. Baker (Danville, PA, USA). MS grade ethyl acetate and formic acid were purchased from Sigma-Aldrich Laboratories, Inc. (St. Louis, MO, USA). Rhizomes of *Smilax glabra* Roxb. were obtained from Chengdu's Hehuachi Herbal Medicine Market.

### 3.2. Preparation of Standards and Samples

Stock solutions of the five standards, astilbin, isoastilbin, neoastilbin, neoisoastilbin, and engeletin, were prepared in acetonitrile at the concentration of 1 mg/mL, and then diluted to 1 μg/mL with 0.1% acetic acid-containing acetonitrile. Powdered rhizomes of *Smilax glabra* (5 g) were extracted three times with 100 mL of 60% aqueous methanol for 1 h each time under ultrasound at room temperature. The combined extracts were concentrated to 100 mL, and then consecutively partitioned with petroleum ether and ethyl acetate. The ethyl acetate extract (1 mg) was dissolved in 1 mL of acetonitrile and then diluted to 10 μg/mL with 0.1% acetic acid-containing acetonitrile, followed by centrifugation at 15,000 rpm/min prior to LC/MS analysis.

### 3.3. UHPLC-Q-TOF-MS Analysis

An Agilent 1290 Ultra-high Performance Liquid Chromatography (UHPLC, Agilent Technologies, Santa Clara, CA, USA) consisting of an autosampler, thermostatted column compartment and binary pump and equipped with an Eclipse XDB-C18 column (2.1 × 100 mm, 1.8 μm, Agilent Technologies) was employed for the separation of components. The column temperature was maintained at 40 °C. The injection volume was 1 μL. The mobile phase comprised of 0.1% acetic acid (A) and 0.1% acetic acid-containing acetonitrile (B) with the following gradient, 0–1 min, 5% B; 1–1.5 min, 5% to 15% B; 1.5–54 min, 15% to 25% B; 54–55 min, 25% to 95% B; 55–57 min, 95% B; 57–57.1 min, return to 5% B. The mass spectrometry was conducted on a 6550 UHD Accurate-Mass Q-TOF/MS system (Agilent Technologies) with a dual Agilent Jet Stream electrospray ion source (dual AJS ESI). The negative mode was applied due to high response and sensitivity. The MS parameters were optimized by using the standard of astilbin and set as follows: gas temperature at 250 °C, drying gas flow at 15 L/min, nebulizer pressure at 25 psig, sheath gas temperature at 300 °C, sheath gas flow at 11 L/min, capillary voltage at 3500 V, and nozzle voltage at 2000 V. The mass spectra were recorded across the range of 100–1700 *m/z* for qualitative analyses. The data were processed with the MassHunter Workstation Data Acquisition Software and Agilent Metabolite ID Software. Accurate mass measurements of each peak were obtained by using a low flow of TOF reference mixture, containing the internal reference masses at *m/z* 119.0363 (C_5_H_4_N_4_) and 966.0007 (C_19_H_20_F_24_N_3_O_8_P_3_). For MS/MS acquisition, automated and target MS/MS were applied and the collision cell energy was set at 30 eV.

### 3.4. Establishment of the Databases

It was reported that flavanonols and flavanols are the major constituents in *S. glabra* [8,9,10], thus based on the principles of flavonoid biosynthesis, e.g., hydroxylation, methylation, phenylpropanoid-substitution, glycosylation, and so on, two databases were established to identify the flavanonols and flavanols, respectively, using naringenin and catechin as core compounds. The databases were established utilizing the MassHunter Metabolite ID software (Agilent Technologies, Inc., Version B.04.00). The parameters of Agilent MassHunter Metabolite ID software were set as follows: the criteria of metabolite identification including “molecular feature extractor”, “isotopic pattern matching”, “EIC compound search” and “mass defect filter” were selected and the metabolites were identified if the total relevance exceeded 75%. The relative tolerance for MS mass accuracy and retention time was set 10 ppm and 1.00%, respectively. In “find compounds by molecular features”, the peaks from 4 to 50 min with heights over 200 counts were generated and the accurate mass recording was restricted across the *m*/*z* 200–1000 range in negative ion mode for one charge state. Moreover, the expected data variation of mass and isotope abundance between the calculated and observed values were set for 2.0 mDa + 5.6 ppm and 7.5%. Other parameters in Metabolite ID were the default settings.

## 4. Conclusions

In conclusion, a quick and automatic flavonoid profiling approach was established based on the flavonoid biosynthetic pathways utilizing a commercial metabolite software package. By comparison of acquired mass spectra with that of predicted compounds in the database, 624 flavonoids were automatically searched and preliminarily determined from the rhizomes of *Smilax glabra*. Among them, 104 compounds with higher scores were definitely or tentatively identified by corresponding fragmentation ions in the MS/MS spectra and/or comparison of the retention times with the reference standards. Because the possible structures in the herb were predicted in the database according to known biotransformation principles, the secondary metabolites, even in trace amounts, could be searched. At the same time, the possible structures were automatically provided, which speeded up the structural elucidation, while for the potential new compounds, professional experience and intelligence were still needed. This approach was limited for the identification of some isomers. Nevertheless, this method can rapidly provide clues about the possible components in the plants by the combination of software-assisted plant secondary metabolite database and UHPLC-Q-TOF-MS analysis. This platform should also be valuable for discovering new flavonoids in other plants as a promising source for drug discovery.

## Supplementary Materials

Supplementary materials can be accessed at: http://www.mdpi.com/1420-3049/20/03/3955/s1.
