# Supplementary material for: Metabolites Software-Assisted Flavonoid Hunting in Plants Using Ultra-High Performance Liquid Chromatography-Quadrupole-Time of Flight Mass Spectrometry
Source: Molecules. 2015 Mar 2;20(3):3955–71. doi: 10.3390/molecules20033955 (PMC6272731; doi:10.3390/molecules20033955)
Supplement: Supplementary file 1 [file molecules-20-03955-s001.pdf]

Supplementary Materials

Table S1. Identified compounds from *Smilax glabra* using flavonoid databases and UHPLC-Q-TOF-MS analysis.

No.	Metabolite ID Matching: RT (min)	Formula $[M-H]^-$	Isotopic Peak No.	Calculated Mass	Observed Mass	Mass Diff. (ppm)	Calculated Abundance	Observed Abundance	Relative Error (%)	MS/MS Fragment Ion	Proposed Structure
**1**	15.1	$C_{15}H_{11}O_6$	1	287.0561	287.0569	−2.62	100	100	0	125.0240,151.0033	Dihydrokaempferol or isomer
			2	288.0595	288.0598	−0.96	16.6	14.3	2.3		
			3	289.0617	289.062	−1.34	2.5	2.4	0.1		
**2**	26.9	$C_{15}H_{11}O_6$	1	287.0561	287.0560	0.35	100	100	0	125.0243, 151.0041	Dihydrokaempferol or isomer
			2	288.0595	288.0594	0.45	16.6	15.1	1.5		
			3	289.0617	289.0637	−7.15	2.5	2	0.5		
**3**	40.8	$C_{15}H_{11}O_5$	1	271.0612	271.0621	−3.31	100	100	0	119.0523, 151.0060	Naringenin
			2	272.0646	272.0647	−0.6	16.5	14.5	2		
			3	273.0668	273.0668	0.34	2.3	1.7	0.6		
**4**	9.9	$C_{21}H_{21}O_{11}$	1	449.1089	449.1093	−0.89	100	100	0	285.0401, 151.0032	Neoastilbin
			2	450.1123	450.1104	4.21	23.4	30.5	7.1		
			3	451.1146	451.1133	2.86	4.9	6.4	1.5		
			4	452.1172	452.1158	3.26	0.7	0.9	0.2		
**5**	10.6	$C_{21}H_{21}O_{11}$	1	449.1089	449.1107	−3.87	100	100	0	285.0404, 151.0036	Astilbin
			2	450.1123	450.1115	1.88	23.4	29.9	6.5		
			3	451.1146	451.1137	1.88	4.9	6.5	1.6		
			4	452.1172	452.1162	2.36	0.7	0.9	0.2		
**6**	14.8	$C_{21}H_{21}O_{11}$	1	449.1089	449.1084	1.14	100	100	0	285.0411, 151.0038	Neoisoastilbin
			2	450.1123	450.11	5.16	23.4	27.7	4.3		
			3	451.1146	451.1129	3.64	4.9	5.4	0.5		
			4	452.1172	452.1145	6.1	0.7	0.7	0		
**7**	15.9	$C_{21}H_{21}O_{11}$	1	449.1089	449.1107	−3.91	100	100	0	285.0406, 151.0037	Isoastilbin
			2	450.1123	450.114	−3.69	23.4	21	2.4		
			3	451.1146	451.1134	2.63	4.9	5.8	0.9		
			4	452.1172	452.1136	8.16	0.7	1	0.3		

Table S1. *Cont.*

No.	Metabolite ID Matching									MS/MS Fragment Ion	Proposed Structure
	RT (min)	Formula $[M-H]^-$	Isotopic Peak No.	Calculated Mass	Observed Mass	Mass Diff. (ppm)	Calculated Abundance	Observed Abundance	Relative Error (%)		
**8**	16.6	$C_{21}H_{21}O_{10}$	1	433.114	433.1157	−3.97	100	100	0	287.0559, 269.0460, 259.0609	Neoengeletin
			2	434.1174	434.1165	2.16	23.3	30.0	6.7		
			3	435.1197	435.1188	2.12	4.7	6	1.3		
			4	436.1224	436.1211	2.9	0.7	0.8	0.1		
**9**	19.2	$C_{21}H_{21}O_{10}$	1	433.114	433.1158	−4.1	100	100	0	287.0556, 269.0461, 259.0614	Engeletin
			2	434.1174	434.1188	−3.19	23.3	20.8	2.5		
			3	435.1197	435.1204	−1.55	4.7	3.9	0.8		
			4	436.1224	436.1222	0.48	0.7	0.5	0.2		
**10**	20.9	$C_{21}H_{21}O_{10}$	1	433.114	433.1155	−3.48	100	100	0	287.0561, 269.0455, 259.0612	Neoisoengeletin
			2	434.1174	434.1196	−4.93	23.3	21	2.3		
			3	435.1197	435.121	−2.99	4.7	3.8	0.9		
			4	436.1224	436.123	−1.34	0.7	0.6	0.1		
**11**	24.6	$C_{21}H_{21}O_{10}$	1	433.114	433.1160	−4.56	100	100	0	287.0570, 269.0466 259.0622	Isoengeletin
			2	434.1174	434.1183	−2.1	23.3	36.1	12.8		
			3	435.1197	435.1188	1.98	4.7	7.2	2.5		
			4	436.1224	436.122	0.77	0.7	0.9	0.2		
**12**	9.5	$C_{15}H_{11}O_7$	1	303.051	303.0500	3.34	100	100	0	285.0387, 125.0218	Taxifolin
			2	304.0544	304.0531	4.34	16.6	16.5	0.1		
			3	305.0565	305.0551	4.33	2.7	2.4	0.3		
			4	306.0592	306.0575	5.34	0.3	0.2	0.1		
**13**	6.1	$C_{30}H_{29}O_{15}$	1	629.1512	629.1511	0.11	100	100	0	475.1258, 449.1096, 303.0511, 285.0406, 177.0190, 151.0032	β-Dihydroxyphenyl-α-carboxyl-3-oxopropyl-substituted neoastilbin
			2	630.1546	630.1541	0.71	33.4	30.9	2.5		
			3	631.1571	631.1554	2.67	8.5	7.3	1.2		
			4	632.1597	632.1568	4.6	1.6	1.3	0.3		

Table S1. *Cont.*

No.	Metabolite ID Matching									MS/MS Fragment Ion	Proposed Structure
	RT (min)	Formula $[M-H]^-$	Isotopic Peak No.	Calculated Mass	Observed Mass	Mass Diff. (ppm)	Calculated Abundance	Observed Abundance	Relative Error (%)		
**14**	6.3	$C_{30}H_{29}O_{15}$	1	629.1512	629.1508	0.58	100	100	0	-	β-Dihydroxyphenyl-α-carboxyl-3-oxopropyl-substituted neoastilbin
			2	630.1546	630.1532	2.14	33.4	30.5	2.9		
			3	631.1571	631.1547	3.7	8.5	7.1	1.4		
			4	632.1597	632.1559	6.12	1.6	1.3	0.3		
**15**	7.1	$C_{30}H_{29}O_{15}$	1	629.1512	629.1515	−0.46	100	100	0	475.1251, 449.1093, 303.0511, 285.0405, 177.0189,151.0030	β-Dihydroxyphenyl-α-carboxyl-3-oxopropyl-substituted astilbin
			2	630.1546	630.1555	−1.47	33.4	31.4	2		
			3	631.1571	631.1578	−1.13	8.5	7.1	1.4		
			4	632.1597	632.1596	0.29	1.6	1.2	0.4		
**16**	7.2	$C_{30}H_{29}O_{15}$	1	629.1512	629.1517	−0.75	100	100	0	475.1265, 449.1104, 303.0514, 285.0409, 177.0188, 151.0028	β-Dihydroxyphenyl-α-carboxyl-3-oxopropyl-substituted astilbin
			2	630.1546	630.1561	−2.4	33.4	30.9	2.5		
			3	631.1571	631.1574	−0.52	8.5	7.5	1		
			4	632.1597	632.158	2.7	1.6	1.4	0.2		
**17**	8.9	$C_{30}H_{29}O_{15}$	1	629.1512	629.1499	2.09	100	100	0	-	β-Dihydroxyphenyl-α-carboxyl-3-oxopropyl-substituted neoisoastilbin
			2	630.1546	630.1544	0.37	33.4	31.4	2		
			3	631.1571	631.1573	−0.36	8.5	8	0.5		
			4	632.1597	632.1545	8.25	1.6	1.5	0.1		
**18**	9.3	$C_{30}H_{29}O_{15}$	1	629.1512	629.1511	0.08	100	100	0	-	β-Dihydroxyphenyl-α-carboxyl-3-oxopropyl-substituted neoisoastilbin
			2	630.1546	630.1539	1.16	33.4	31.2	2.2		
**19**	12.0	$C_{30}H_{29}O_{15}$	1	629.1512	629.1519	−1.12	100	100	0	-	β-Dihydroxyphenyl-α-carboxyl-3-oxopropyl-substituted isoastilbin
			2	630.1546	630.1548	−0.32	33.4	31.9	1.5		
			3	631.1571	631.1549	3.5	8.5	9.7	1.2		
			4	632.1597	632.1555	6.66	1.6	2.4	0.8		

Table S1. *Cont.*

No.	Metabolite ID Matching									MS/MS Fragment Ion	Proposed Structure
	RT (min)	Formula $[M–H]^-$	Isotopic Peak No.	Calculated Mass	Observed Mass	Mass Diff. (ppm)	Calculated Abundance	Observed Abundance	Relative Error (%)		
**20**	13.0	$C_{30}H_{29}O_{15}$	1	629.1512	629.1511	0.22	100	100	0	-	β-Dihydroxyphenyl-α-carboxyl-3-oxopropyl-substituted isoastilbin
			2	630.1546	630.1543	0.42	33.4	33.3	0.1		
			3	631.1571	631.1547	3.78	8.5	9	0.5		
			4	632.1597	632.1559	6.02	1.6	2.4	0.8		
**21**	21.9	$C_{30}H_{27}O_{14}$	1	611.1406	611.1418	−1.86	100	100	0	-	Dihydroxyphenylpropanoid-substituted astilbin or isomer
			2	612.144	612.1444	−0.58	33.3	32.5	0.8		
			3	613.1465	613.1461	0.68	8.2	7.2	1		
			4	614.1492	614.1476	2.64	1.5	1.3	0.2		
**22**	23.3	$C_{30}H_{27}O_{14}$	1	611.1406	611.1392	2.31	100	100	0	-	Dihydroxyphenylpropanoid-substituted astilbin or isomer
			2	612.144	612.1417	3.84	33.3	26.3	7		
**23**	29.5	$C_{30}H_{27}O_{14}$	1	611.1406	611.1427	−3.33	100	100	0	465.0822, 447.0724, 355.0445, 337.0321, 327.0497	Dihydroxyphenylpropanoid-substituted astilbin or isomer
			2	612.144	612.1451	−1.77	33.3	29	4.3		
			3	613.1465	613.1453	2.07	8.2	7.1	1.1		
			4	614.1492	614.1452	6.47	1.5	1.5	0		
**24**	31.5	$C_{30}H_{27}O_{14}$	1	611.1406	611.1388	3.06	100	100	0	465.0815, 447.0703, 355.0439, 337.0335, 327.0509, 202,9945	Dihydroxyphenylpropanoid-substituted astilbin or isomer
			2	612.144	612.1419	3.48	33.3	28.8	4.5		
			3	613.1465	613.1434	5.05	8.2	10.8	2.6		
			4	614.1492	614.1438	8.83	1.5	2.1	0.6		
**25**	33.7	$C_{30}H_{27}O_{14}$	1	611.1406	611.1388	2.91	100	100	0	465.0822, 447.0708, 355.0462, 337.0343, 327.0508, 202.9989	Dihydroxyphenylpropanoid-substituted astilbin or isomer
			2	612.144	612.1422	2.99	33.3	32.4	0.9		
			3	613.1465	613.1402	10.33	8.2	12.7	4.5		
			4	614.1492	614.1408	13.71	1.5	4.3	2.8		

Table S1. *Cont.*

No.	Metabolite ID Matching									MS/MS Fragment Ion	Proposed Structure
	RT (min)	Formula $[M-H]^-$	Isotopic Peak No.	Calculated Mass	Observed Mass	Mass Diff. (ppm)	Calculated Abundance	Observed Abundance	Relative Error (%)		
**26**	43.8	$C_{30}H_{27}O_{14}$	1	611.1406	611.1393	2.1	100	100	0	465.0807, 447.0699, 355.0444, 337.0330, 327.0513, 202.9976	Dihydroxyphenylpropanoid-substituted astilbin or isomer
			2	612.144	612.142	3.23	33.3	29.4	3.9		
			3	613.1465	613.1399	14.12	8.2	15.2	7		
			4	614.1492	614.1398	15.27	1.5	4.5	3		
**27**	11.7	$C_{27}H_{31}O_{15}$	1	595.1668	595.1679	−1.73	100	100	0	–	Neoengeletin glucoside
			2	596.1702	596.1717	−2.41	30.1	27.9	2.2		
			3	597.1726	597.1714	1.97	7.5	8.1	0.6		
			4	598.1753	598.1726	4.43	1.3	1.4	0.1		
**28**	11.9	$C_{27}H_{31}O_{15}$	1	595.1668	595.1672	−0.52	100	100	0	–	Neoengeletin glucoside
			2	596.1702	596.1696	1.07	30.1	29.2	0.9		
			3	597.1726	597.1679	7.83	7.5	8.4	0.9		
**29**	12.3	$C_{27}H_{31}O_{15}$	1	595.1668	595.1689	−3.41	100	100	0	–	Engeletin glucoside
			2	596.1702	596.171	−1.28	30.1	27.1	3		
			3	597.1726	597.1672	9.02	7.5	13.8	6.3		
			4	598.1753	598.1712	6.76	1.3	2.8	1.5		
**30**	12.7	$C_{27}H_{31}O_{15}$	1	595.1668	595.1671	−0.48	100	100	0	–	Engeletin glucoside
			2	596.1702	596.1696	1.03	30.1	34.8	4.7		
			3	597.1726	597.164	14.42	7.5	15.3	7.8		
			4	598.1753	598.1644	18.18	1.3	4.2	2.9		
**31**	14.6	$C_{27}H_{31}O_{15}$	1	595.1668	595.1678	−1.63	100	100	0	–	Neoisoengeletin glucoside
			2	596.1702	596.1716	−2.23	30.1	29.3	0.8		
			3	597.1726	597.1712	2.31	7.5	7.2	0.3		
			4	598.1753	598.169	10.49	1.3	1.9	0.6		

Table S1. *Cont.*

No.	Metabolite ID Matching									MS/MS Fragment Ion	Proposed Structure
	RT (min)	Formula $[M–H]^-$	Isotopic Peak No.	Calculated Mass	Observed Mass	Mass Diff. (ppm)	Calculated Abundance	Observed Abundance	Relative Error (%)		
**32**	15	$C_{27}H_{31}O_{15}$	1	595.1668	595.168	−1.87	100	100	0	–	Neoisoengeletin glucoside
			2	596.1702	596.1717	−2.43	30.1	28	2.1		
			3	597.1726	597.1681	7.55	7.5	10.9	3.4		
			4	598.1753	598.1691	10.4	1.3	2.1	0.8		
**33**	16.9	$C_{27}H_{31}O_{15}$	1	595.1668	595.1698	−4.92	100	100	0	–	Isoengeletin glucoside
			2	596.1702	596.1722	−3.32	30.1	28.2	1.9		
			3	597.1726	597.1715	1.9	7.5	8.1	0.6		
**34**	3.8	$C_{15}H_{13}O_6$	1	289.0718	289.0728	−3.59	100	100	0	245.0796, 205.0490, 179.0341	Catechin
			2	290.0753	290.0752	−0.27	16.6	20.1	3.5		
			3	291.0733	291.0778	−1.71	2.5	2.8	0.3		
			4	292.08	292.0806	−1.94	0.3	0.3	0		
**35**	4.8	$C_{15}H_{13}O_6$	1	289.0718	289.0724	−2.23	100	100	0	245.0818, 205.0505, 179.0350	Epicatechin
			2	290.0753	290.0761	−3.2	16.6	15.5	1.1		
			3	291.0733	291.0786	−4.28	2.5	2.2	0.3		
			4	292.08	292.0811	−3.76	0.3	0.2	0.1		
**36**	3.2	$C_{30}H_{25}O_{12}$	1	577.1351	577.1359	−1.31	100	100	0	451.1042, 425.0881, 407.0773, 289.0718, 125.0239	Procyanidin B or isomer
			2	578.1385	578.1402	−2.79	33.2	30.7	2.5		
			3	579.1411	579.1419	−1.31	7.8	6.3	1.5		
			4	580.1438	580.1443	−0.8	1.4	1	0.4		
**37**	3.5	$C_{30}H_{25}O_{12}$	1	577.1351	577.1353	−0.26	100	100	0	451.1039, 425.0882, 407.0775, 289.0716, 125.0234	Procyanidin B or isomer
			2	578.1385	578.1391	−1.02	33.2	30.8	2.4		
			3	579.1411	579.141	0.24	7.8	6.5	1.3		
			4	580.1438	580.1434	0.78	1.4	1	0.4		
**38**	3.7	$C_{30}H_{25}O_{12}$	1	577.1351	577.1332	3.33	100	100	0	-	Procyanidin B or isomer
			2	578.1385	578.1359	4.57	33.2	31.7	1.5		

Table S1. *Cont.*

No.	Metabolite ID Matching									MS/MS Fragment Ion	Proposed Structure
	RT (min)	Formula $[M-H]^-$	Isotopic Peak No.	Calculated Mass	Observed Mass	Mass Diff. (ppm)	Calculated Abundance	Observed Abundance	Relative Error (%)		
**39**	3.8	$C_{30}H_{25}O_{12}$	1	577.1351	577.1366	−2.56	100	100	0	-	Procyanidin B or isomer
			2	578.1385	578.1392	−1.13	33.2	30.8	1.4		
**40**	4.1	$C_{30}H_{25}O_{12}$	1	577.1351	577.1378	−4.61	100	100	0	407.0770, 289.0715, 125.0233	Procyanidin B or isomer
			2	578.1385	578.1408	−3.91	33.2	30.9	2.3		
			3	579.1411	579.1458	−7.99	7.8	9.7	1.9		
			4	580.1438	580.1504	−11.36	1.4	1.9	0.5		
**41**	4.8	$C_{30}H_{25}O_{12}$	1	577.1351	577.1374	−3.88	100	100	0	-	Procyanidin B or isomer
			2	578.1385	578.1405	−3.42	33.2	31.1	1.1		
**42**	5.2	$C_{30}H_{25}O_{12}$	1	577.1351	577.1339	2.23	100	100	0	-	Procyanidin B or isomer
			2	578.1385	578.1392	−1.2	33.2	30.9	2.3		
			3	579.1411	579.1466	−9.45	7.8	12.2	4.4		
**43**	5.8	$C_{30}H_{25}O_{12}$	1	577.1351	577.1364	−2.09	100	100	0	407.0768, 289.0713, 125.0230	Procyanidin B or isomer
			2	578.1385	578.1394	−1.54	33.2	31.4	1.8		
			3	579.1411	579.1495	−14.37	7.8	27.3	19.5		
			4	580.1438	580.1538	−17.13	1.4	7.2	5.8		
**44**	6.0	$C_{30}H_{25}O_{12}$	1	577.1351	577.1372	−3.60	100	100	0	407.0765, 289.0710, 125.0228	Procyanidin B or isomer
			2	578.1385	578.1410	−4.25	33.2	30.1	2.1		
			3	579.1411	579.1430	−3.23	7.8	6.9	0.9		
			4	580.1438	580.1453	−2.67	1.4	1.2	0.2		
**45**	7.2	$C_{30}H_{25}O_{12}$	1	577.1351	577.1327	4.3	100	100	0	-	Procyanidin B or isomer
			2	578.1385	577.1359	4.56	33.2	34.7	1.5		
			3	579.1411	579.1321	15.67	7.8	13.8	6		
**46**	8	$C_{30}H_{25}O_{12}$	1	577.1351	577.1367	−2.66	100	100	0	-	Procyanidin B or isomer
			2	578.1385	578.1412	−4.66	33.2	24.4	8.8		
			3	579.1411	579.1434	−3.87	7.8	10	2.2		

Table S1. *Cont.*

No.	Metabolite ID Matching									MS/MS Fragment Ion	Proposed Structure
	RT (min)	Formula $[M-H]^-$	Isotopic Peak No.	Calculated Mass	Observed Mass	Mass Diff. (ppm)	Calculated Abundance	Observed Abundance	Relative Error (%)		
**47**	22.6	$C_{30}H_{25}O_{12}$	1	577.1351	577.1366	−2.54	100	100	0	-	Procyanidin B or isomer
			2	578.1385	578.1391	−0.89	33.2	30.1	3.1		
			3	579.1411	579.1418	−1.22	7.8	5.2	2.6		
**48**	24	$C_{30}H_{25}O_{12}$	1	577.1351	577.1367	−2.61	100	100	0	-	Procyanidin B or isomer
			2	578.1385	578.1393	−1.32	33.2	35.5	2.3		
			3	579.1411	579.1395	2.82	7.8	4.4	3.3		
**49**	25.1	$C_{30}H_{25}O_{12}$	1	577.1351	577.1377	−4.5	100	100	0	-	Procyanidin B or isomer
			2	578.1385	578.1381	0.74	33.2	32.6	0.6		
			3	579.1411	579.1415	−0.64	7.8	8.1	0.3		
**50**	35.7	$C_{30}H_{25}O_{12}$	1	577.1351	577.1363	−1.96	100	100	0	-	Procyanidin B or isomer
			2	578.1385	578.1397	−2.04	33.2	37.9	4.5		
			3	579.1411	579.15	−15.25	7.8	7.3	0.5		
			4	580.1438	580.1382	9.73	1.4	1.4	0		
**51**	37.6	$C_{30}H_{25}O_{12}$	1	577.1351	577.1365	−2.42	100	100	0	-	Procyanidin B or isomer
			2	578.1385	578.1377	1.48	33.2	31.1	1.1		
			3	579.1411	579.138	5.47	7.8	5.8	2		
**52**	3.8	$C_{30}H_{25}O_{11}$	1	561.1402	561.1422	−3.5	100	100	0	435.1101, 407.0784, 289.0721, 125.0241	Tetrahydroxyflavanol (4→8)-catechin or isomer
			2	562.1436	562.1449	−2.28	33.2	30.8	2.4		
			3	563.1463	563.1472	−1.59	5.3	6.5	0.8		
			4	564.1489	564.1497	−1.26	0.9	1.1	0.2		
**53**	3.9	$C_{30}H_{25}O_{11}$	1	561.1402	561.141	−1.43	100	100	0	435.1085, 407.0779, 289.0722, 125.0241	Tetrahydroxyflavanol (4→8)-catechin or isomer
			2	562.1436	562.1446	−1.67	33.2	31.2	2		
			3	563.1463	563.1465	−0.36	5.3	6.4	1.1		
			4	564.1489	564.1483	1.16	0.9	1.1	0.2		

Table S1. *Cont.*

No.	Metabolite ID Matching									MS/MS Fragment Ion	Proposed Structure
	RT (min)	Formula $[M-H]^-$	Isotopic Peak No.	Calculated Mass	Observed Mass	Mass Diff. (ppm)	Calculated Abundance	Observed Abundance	Relative Error (%)		
**54**	4.4	$C_{30}H_{25}O_{11}$	1	561.1402	561.1394	1.49	100	100	0	435.1046, 407.0734, 289.0675, 125.0196	Tetrahydroxyflavanol (4→8)-catechin or isomer
			2	562.1436	562.1436	−0.01	33.2	30.5	2.7		
			3	563.1463	563.1453	1.72	5.3	6.3	1		
			4	564.1489	564.1473	2.97	0.9	1	0.1		
**55**	4.6	$C_{30}H_{25}O_{11}$	1	561.1402	561.1379	1.04	100	100	0	-	Polymer of tetrahydroxyflavanol and catechin
			2	562.1436	562.1426	1.75	33.2	33.3	0.1		
			3	563.1463	563.144	3.94	5.3	8.7	3.4		
			4	564.1489	564.146	5.31	0.9	1.7	0.8		
**56**	4.8	$C_{30}H_{25}O_{11}$	1	561.1402	561.1421	−3.33	100	100	0	-	Polymer of tetrahydroxyflavanol and catechin
			2	562.1436	562.1452	−2.78	33.2	29.9	3.3		
			3	563.1463	563.1471	−1.4	5.3	6.3	1		
			4	564.1489	564.1507	−3.14	0.9	1.1	0.2		
**57**	4.9	$C_{30}H_{25}O_{11}$	1	561.1402	561.1379	1.03	100	100	0	-	Polymer of tetrahydroxyflavanol and catechin
			2	562.1436	562.1429	1.26	33.2	32.6	0.6		
			3	563.1463	563.1456	1.17	5.3	9.2	3.9		
			4	564.1489	564.1474	2.81	0.9	1.4	0.5		
**58**	5.6	$C_{30}H_{25}O_{11}$	1	561.1402	561.1411	−1.6	100	100	0	-	Polymer of tetrahydroxyflavanol and catechin
			2	562.1436	562.144	−0.7	33.2	31.3	0.9		
			3	563.1463	563.1468	−1	5.3	7.7	2.4		
			4	564.1489	564.152	−5.37	0.9	1.2	0.3		
**59**	6.7	$C_{30}H_{25}O_{11}$	1	561.1402	561.1424	−3.8	100	100	0	-	Polymer of tetrahydroxyflavanol and catechin
			2	562.1436	562.1454	−3.09	33.2	29.5	3.7		
			3	563.1463	563.1477	−2.5	5.3	6.7	1.4		
			4	564.1489	564.1494	−0.78	0.9	1.2	0.3		

Table S1. *Cont.*

No.	Metabolite ID Matching									MS/MS Fragment Ion	Proposed Structure
	RT (min)	Formula $[M-H]^-$	Isotopic Peak No.	Calculated Mass	Observed Mass	Mass Diff. (ppm)	Calculated Abundance	Observed Abundance	Relative Error (%)		
**60**	7.1	$C_{30}H_{25}O_{11}$	1	561.1402	561.1389	2.4	100	100	0	-	Polymer of tetrahydroxyflavanol and catechin
			2	562.1436	562.1414	4.02	33.2	27.6	5.6		
			3	563.1463	563.142	7.5	5.3	6.2	0.9		
**61**	7.7	$C_{30}H_{25}O_{11}$	1	561.1402	561.1405	−0.55	100	100	0	-	Polymer of tetrahydroxyflavanol and catechin
			2	562.1436	562.1444	−1.33	33.2	33.8	0.6		
			3	563.1463	563.1498	−6.28	5.3	8.4	3.1		
			4	564.1489	564.1484	0.95	0.9	2.4	1.5		
**62**	7.9	$C_{30}H_{25}O_{11}$	1	561.1402	561.1415	−2.25	100	100	0	-	Polymer of tetrahydroxyflavanol and catechin
			2	562.1436	562.1438	−0.24	33.2	30.2	3		
			3	563.1463	563.1468	−0.98	5.3	20.9	15.6		
			4	564.1489	564.1458	5.52	0.9	5.6	4.7		
**63**	8.5	$C_{30}H_{25}O_{11}$	1	561.1402	561.1413	−1.88	100	100	0	-	Polymer of tetrahydroxyflavanol and catechin
			2	562.1436	562.1441	−0.93	33.2	30.3	2.9		
			3	563.1463	563.1469	−1.21	5.3	6.2	0.9		
			4	564.1489	564.1492	−0.49	0.9	1.2	0.3		
**64**	8.8	$C_{30}H_{25}O_{11}$	1	561.1402	561.1402	0.13	100	100	0	-	Polymer of tetrahydroxyflavanol and catechin
			2	562.1436	562.1429	1.28	33.2	31.7	1.5		
			3	563.1463	563.145	2.26	5.3	7.2	1.9		
			4	564.1489	564.147	3.38	0.9	1.2	0.3		
**65**	10.2	$C_{30}H_{25}O_{11}$	1	561.1402	561.1397	0.94	100	100	0	-	Polymer of tetrahydroxyflavanol and catechin
			2	562.1436	562.1439	−0.51	33.2	31.9	1.3		
			3	563.1463	563.1458	0.77	5.3	12.1	6.8		

Table S1. *Cont.*

No.	Metabolite ID Matching									MS/MS Fragment Ion	Proposed Structure
	RT (min)	Formula $[M-H]^-$	Isotopic Peak No.	Calculated Mass	Observed Mass	Mass Diff. (ppm)	Calculated Abundance	Observed Abundance	Relative Error (%)		
**66**	9.2	$C_{24}H_{19}O_9$	1	451.1035	451.105	−3.51	100	100	0	341.0657, 217.0215	Cinchonain Ia or isomer
			2	452.1068	452.107	−0.44	26.5	30	3.5		
			3	453.1093	453.1105	−2.48	5.2	5.3	0.1		
			4	454.112	454.1123	−0.59	0.8	0.7	0.1		
**67**	15.2	$C_{24}H_{19}O_9$	1	451.1035	451.1055	−4.57	100	100	0	341.0654, 217.0130	Cinchonain Ia or isomer
			2	452.1068	452.1065	0.86	26.5	33.6	7.1		
			3	453.1093	453.109	0.74	5.2	6.4	1.2		
			4	454.112	454.1118	0.48	0.8	0.9	0.1		
**68**	15.4	$C_{24}H_{19}O_9$	1	451.1035	451.1035	−0.12	100	100	0	341.0643, 217.0128	Cinchonain Ia or isomer
			2	452.1068	452.1048	4.55	26.5	33.8	7.3		
			3	453.1093	453.1077	3.59	5.2	6.1	0.9		
			4	454.112	454.1103	3.83	0.8	0.9	0.1		
**69**	15.8	$C_{24}H_{19}O_9$	1	451.1035	451.1046	−2.56	100	100	0	341.0582, 217.0095	Cinchonain Ia or isomer
			2	452.1068	452.1061	1.56	26.5	29.4	2.9		
			3	453.1093	453.1094	−0.22	5.2	5.2	0		
			4	454.112	454.1109	2.48	0.8	0.7	0.1		
**70**	20.5	$C_{24}H_{19}O_9$	1	451.1035	451.1046	−2.56	100	100	0	341.0678, 217.0147	Cinchonain Ia or isomer
			2	452.1068	452.1061	1.56	26.5	29.4	2.9		
			3	453.1093	453.1094	−0.22	5.2	5.2	0		
			4	454.112	454.1109	2.48	0.8	0.7	0.1		
**71**	20.9	$C_{24}H_{19}O_9$	1	451.1035	451.1037	−0.62	100	100	0	341.0673, 217.0146	Cinchonain Ia or isomer
			2	452.1068	452.1071	−0.63	26.5	25.1	1.4		
			3	453.1093	453.1099	−1.25	5.2	4.3	0.9		
			4	454.112	454.1121	−0.28	0.8	0.6	0.2		

Table S1. *Cont.*

No.	Metabolite ID Matching									MS/MS Fragment Ion	Proposed Structure
	RT (min)	Formula $[M-H]^-$	Isotopic Peak No.	Calculated Mass	Observed Mass	Mass Diff. (ppm)	Calculated Abundance	Observed Abundance	Relative Error (%)		
**72**	28.9	$C_{24}H_{19}O_9$	1	451.1035	451.1045	−2.35	100	100	0	341.0676, 217.0149	Cinchonain Ia or isomer
			2	452.1068	452.1079	−2.25	26.5	25.4	1.1		
			3	453.1093	453.1106	−2.75	5.2	4.2	1		
			4	454.112	454.1123	−0.7	0.8	0.6	0.2		
**73**	30.4	$C_{24}H_{19}O_9$	1	451.1035	451.105	−3.39	100	100	0	341.0682, 217.0150	Cinchonain Ia or isomer
			2	452.1068	452.108	−2.5	26.5	25.6	0.9		
			3	453.1093	453.1111	−3.92	5.2	4.4	0.8		
			4	454.112	454.1129	−1.94	0.8	0.6	0.2		
**74**	13.7	$C_{24}H_{19}O_8$	1	435.1085	435.11	−3.37	100	100	0	325.0730, 307.0618, 281.0824	Dihydroxyphenyl-propanoid-substituted tetrahydroxyflavanol
			2	436.1119	436.1134	−3.4	26.5	23.5	3		
			3	437.1145	437.116	−3.5	5	4.3	0.7		
			4	438.1172	438.1175	−0.69	0.7	0.6	0.1		
**75**	17.3	$C_{24}H_{19}O_8$	1	435.1085	435.1106	−4.63	100	100	0	-	Dihydroxyphenyl-propanoid-substituted tetrahydroxyflavanol or isomer
			2	436.1119	436.1128	−1.99	26.5	23.5	3		
			3	437.1145	437.1146	−0.14	5	4.3	0.7		
			4	438.1172	438.1179	−1.71	0.7	0.6	0.1		
**76**	21.9	$C_{24}H_{19}O_8$	1	435.1085	435.1108	−5.22	100	100	0	-	Dihydroxyphenyl-propanoid-substituted tetrahydroxyflavanol or isomer
			2	436.1119	436.1138	−4.34	26.5	24.5	2		
			3	437.1145	437.1166	−4.74	5	4.9	0.1		
			4	438.1172	438.1181	−2.2	0.7	0.8	0.1		
**77**	22.5	$C_{24}H_{19}O_8$	1	435.1085	435.1101	−3.49	100	100	0	325.0734, 307.0628, 281.0834	Dihydroxyphenyl-propanoid-substituted tetrahydroxyflavanol
			2	436.1119	436.1131	−2.62	26.5	24	2.5		
			3	437.1145	437.1153	−1.93	5	4.4	0.6		
			4	438.1172	438.1168	0.8	0.7	0.6	0.1		

Table S1. *Cont.*

No.	Metabolite ID Matching									MS/MS Fragment Ion	Proposed Structure
	RT (min)	Formula $[M-H]^-$	Isotopic Peak No.	Calculated Mass	Observed Mass	Mass Diff. (ppm)	Calculated Abundance	Observed Abundance	Relative Error (%)		
**78**	23.2	$C_{24}H_{19}O_8$	1	435.1085	435.1101	−3.64	100	100	0	325.0730, 307.0621, 281.0826	Dihydroxyphenyl-propanoid-substituted tetrahydroxyflavanol
			2	436.1119	436.1128	−1.88	26.5	23.9	2.6		
			3	437.1145	437.1152	−1.71	5	4.3	0.7		
			4	438.1172	438.1175	−0.69	0.7	0.5	0.2		
**79**	24.1	$C_{24}H_{19}O_8$	1	435.1085	435.1087	−0.25	100	100	0	-	Dihydroxyphenyl-propanoid-substituted tetrahydroxyflavanol
			2	436.1119	436.1113	1.41	26.5	24.5	2		
			3	437.1145	437.1131	3.11	5	4.5	0.5		
			4	438.1172	438.1167	1.01	0.7	0.7	0		
**80**	39.6	$C_{24}H_{19}O_8$	1	435.1085	435.1107	−5.06	100	100	0	325.0724, 307.0612, 281.0822	Dihydroxyphenyl-propanoid-substituted tetrahydroxyflavanol
			2	436.1119	436.1137	−3.97	26.5	24.8	1.7		
			3	437.1145	437.1161	−3.61	5	4.6	0.4		
			4	438.1172	438.1187	−3.61	0.7	0.7	0		
**81**	40.2	$C_{24}H_{19}O_8$	1	435.1085	435.1088	−0.52	100	100	0	325.0728, 307.0617, 281.0816	Dihydroxyphenyl-propanoid-substituted tetrahydroxyflavanol
			2	436.1119	436.113	−2.39	26.5	24.9	1.6		
			3	437.1145	437.1151	−1.33	5	4.2	0.8		
			4	438.1172	438.1174	−0.52	0.7	0.6	0.1		
**82**	21.6	$C_{33}H_{25}O_{12}$	1	613.1351	613.1384	−5.37	100	100	0	503.1020, 393.0640, 341.0687, 217.0126, 323.0575	4,8,10-Tris(dihydroxy-phenyl)-11-hydroxy-3,4,7,8,11,12-hexahydro-2*H*,6*H*,10*H*-dipyrano[2,3-*f*:2',3'-*h*]-chromene-2,6-dione
			2	614.1385	614.1412	−4.41	36.4	32.9	3.5		
			3	615.1412	615.1449	−5.92	8.9	8.6	0.3		
			4	616.1439	616.1499	−9.67	1.6	1.7	0.1		
**83**	26.1	$C_{33}H_{25}O_{12}$	1	613.1351	613.1365	−2.63	100	100	0	503.0997, 393.0632, 341.0676, 217.0148 323.0569	4,8,10-Tris(dihydroxy-phenyl)-11-hydroxy-3,4,7,8,11,12-hexahydro-2*H*,6*H*,10*H*-dipyrano[2,3-*f*:2',3'-*h*]-chromene-2,6-dione
			2	614.1385	614.1396	−1.8	36.4	36.5	0.1		
			3	615.1412	615.1434	−3.5	8.9	8.1	0.7		
			4	616.1439	616.1448	−1.35	1.6	1.3	0.3		

Table S1. *Cont.*

No.	Metabolite ID Matching									MS/MS Fragment Ion	Proposed Structure
	RT (min)	Formula $[M–H]^−$	Isotopic Peak No.	Calculated Mass	Observed Mass	Mass Diff. (ppm)	Calculated Abundance	Observed Abundance	Relative Error (%)		
**84**	27.6	$C_{33}H_{25}O_{12}$	1	613.1351	613.137	−2.95	100	100	0	503.0989, 393.0622, 341.0668, 217.0142, 323.0564	4,8,10-Tris(dihydroxy-phenyl)-11-hydroxy-3,4,7,8,11,12-hexahydro-2*H*,6*H*,10*H*-dipyrano[2,3-*f*:2',3'-*h*]-chromene-2,6-dione
			2	614.1385	614.1397	−1.83	36.4	36.5	0.1		
			3	615.1412	615.1435	−3.68	8.9	8.1	0.8		
			4	616.1439	616.1449	−1.65	1.6	1.3	0.3		
**85**	33.9	$C_{33}H_{25}O_{12}$	1	613.1351	613.1374	−3.73	100	100	0	503.1012, 393.0636, 341.0684, 217.0130, 323.0571	4,8,10-Tris(dihydroxy-phenyl)-11-hydroxy-3,4,7,8,11,12-hexahydro-2*H*,6*H*,10*H*-dipyrano[2,3-*f*:2',3'-*h*]chromene-2,6-dione
			2	614.1385	614.1404	−2.96	36.4	33.4	3		
			3	615.1412	615.1429	−2.8	8.9	7.9	1		
			4	616.1439	616.1464	−3.96	1.6	1.4	0.2		
**86**	36.8	$C_{33}H_{25}O_{12}$	1	613.1351	613.1364	−1.98	100	100	0	503.1007, 393.0637, 341.0687, 217.0147 323.0571	4,8,10-Tris(dihydroxy-phenyl)-11-hydroxy-3,4,7,8,11,12-hexahydro-2*H*,6*H*,10*H*-dipyrano[2,3-*f*:2',3'-*h*]chromene-2,6-dione
			2	614.1385	614.1495	−1.52	36.4	34.4	2		
			3	615.1412	615.143	−2.86	8.9	7.9	1		
			4	616.1439	616.1451	−1.84	1.6	1.2	0.4		
**87**	38.5	$C_{33}H_{25}O_{12}$	1	613.1351	613.1364	−2.08	100	100	0	503.0988, 393.0624, 341.0674, 217.0142, 323.0564	4,8,10-Tris(dihydroxy-phenyl)-11-hydroxy-3,4,7,8,11,12-hexahydro-2*H*,6*H*,10*H*-dipyrano[2,3-*f*:2',3'-*h*]chromene-2,6-dione
			2	614.1385	614.1396	−1.81	36.4	35	1.4		
			3	615.1412	615.1431	−3.07	8.9	8.2	0.7		
			4	616.1439	616.145	−1.75	1.6	1.3	0.3		
**88**	29.9	$C_{33}H_{25}O_{11}$	1	597.1402	597.1434	−5.25	100	100	0	487.1055, 377.0680, 325.0723, 307.0621	4,8-Bis(dihydroxyphenyl)-10-hydroxyphenyl-11-hydroxy-4,7,8,11,12-penta-hydro-2*H*,6*H*,10*H*-dipyrano[2,3-*f*:2',3'-*h*]chromene-2,6-dione
			2	598.1436	598.1463	−4.51	36.4	33.7	2.7		
			3	599.1464	599.1482	−3.02	8.7	7.2	1.5		
			4	600.1491	600.1514	−3.9	1.6	1.5	0.1		

Table S1. *Cont.*

No.	Metabolite ID Matching									MS/MS Fragment Ion	Proposed Structure
	RT (min)	Formula $[M-H]^-$	Isotopic Peak No.	Calculated Mass	Observed Mass	Mass Diff. (ppm)	Calculated Abundance	Observed Abundance	Relative Error (%)		
**89**	34.6	$C_{33}H_{25}O_{11}$	1	597.1402	597.1438	−5.91	100	100	0	-	4,8-Bis(dihydroxyphenyl)-10-hydroxyphenyl-11-hydroxy-4,7,8,11,12-penta-hydro-2*H*,6*H*,10*H*-dipyrano[2,3-*f*:2',3'-*h*]chromene-2,6-dione or isomer
			2	598.1436	598.1463	−4.4	36.4	31.5	4.9		
			3	599.1464	599.1493	−4.93	8.7	10.4	1.7		
			4	600.1491	600.1469	3.59	1.6	2.9	1.3		
**90**	36.2	$C_{33}H_{25}O_{11}$	1	597.1402	597.1422	−3.29	100	100	0	-	4,8-Bis(dihydroxyphenyl)-10-hydroxyphenyl-11-hydroxy-4,7,8,11,12-penta-hydro-2*H*,6*H*,10*H*-dipyrano[2,3-*f*:2',3'-*h*]chromene-2,6-dione or isomer
			2	598.1436	598.1447	−1.88	36.4	34.4	2		
			3	599.1464	599.1487	−3.88	8.7	10.7	2		
			4	600.1491	600.1492	−0.18	1.6	2.5	0.9		
**91**	37.1	$C_{33}H_{25}O_{11}$	1	597.1402	597.1401	0.18	100	100	0	-	4,8-Bis(dihydroxyphenyl)-10-hydroxyphenyl-11-hydroxy-4,7,8,11,12-penta-hydro-2*H*,6*H*,10*H*-dipyrano[2,3-*f*:2',3'-*h*]chromene-2,6-dione or isomer
			2	598.1436	598.1439	−0.48	36.4	42.2	5.8		
			3	599.1464	599.1489	−4.24	8.7	11.6	2.9		
			4	600.1491	600.1558	−11.19	1.6	2.2	0.6		
**92**	38.5	$C_{33}H_{25}O_{11}$	1	597.1402	597.1424	−3.67	100	100	0	-	4,8-Bis(dihydroxyphenyl)-10-hydroxyphenyl-11-hydroxy-4,7,8,11,12-penta-hydro-2*H*,6*H*,10*H*-dipyrano[2,3-*f*:2',3'-*h*]chromene-2,6-dione or isomer
			2	598.1436	598.1461	−4.12	36.4	35.4	1		
			3	599.1464	599.15	−6.14	8.7	8.6	0.1		
			4	600.1491	600.1443	7.94	1.6	2.3	0.6		

Table S1. *Cont.*

No.	Metabolite ID Matching									MS/MS Fragment Ion	Proposed Structure
	RT (min)	Formula [M–H]$^-$	Isotopic Peak No.	Calculated Mass	Observed Mass	Mass Diff. (ppm)	Calculated Abundance	Observed Abundance	Relative Error (%)		
**93**	41.3	$C_{33}H_{25}O_{11}$	1	597.1402	597.1419	−2.84	100	100	0	-	4,8-Bis(dihydroxyphenyl)-10-hydroxyphenyl-11-hydroxy-4,7,8,11,12-penta-hydro-2*H*,6*H*,10*H*-dipyrano[2,3-*f*:2',3'-*h*]chromene-2,6-dione or isomer
			2	598.1436	598.145	−2.3	36.4	32.3	4.1		
			3	599.1464	599.1465	−0.22	8.7	7.6	1.1		
			4	600.1491	600.1508	−2.96	1.6	1.7	0.1		
**94**	45.7	$C_{33}H_{25}O_{11}$	1	597.1402	597.142	−2.99	100	100	0	-	4,8-Bis(dihydroxyphenyl)-10-hydroxyphenyl-11-hydroxy-4,7,8,11,12-penta hydro-2*H*,6*H*,10*H*-dipyrano[2,3-*f*:2',3'-*h*]chromene-2,6-dione or isomer
			2	598.1436	598.1447	−1.82	36.4	33.1	2.3		
			3	599.1464	599.1497	−5.51	8.7	9	0.3		
			4	600.1491	600.1548	−9.6	1.6	1.9	0.3		
**95**	50	$C_{33}H_{25}O_{11}$	1	597.1402	597.1411	−1.38	100	100	0	-	4,8-Bis(dihydroxyphenyl)-10-hydroxyphenyl-11-hydroxy-4,7,8,11,12-penta hydro-2*H*,6*H*,10*H*-dipyrano[2,3-*f*:2',3'-*h*]chromene-2,6-dione or isomer
			2	598.1436	598.1448	−1.92	36.4	35	1.4		
			3	599.1464	599.1479	−2.62	8.7	9.6	0.9		
			4	600.1491	600.152	−4.94	1.6	2.4	0.8		
**96**	9.1	$C_{26}H_{23}O_{10}$	1	495.1297	495.1301	−0.79	100	100	0	-	Smiglabrone A or isomer
			2	496.1331	496.1336	−1.09	28.8	27.4	1.4		
			3	497.1356	497.1356	0.06	6	5.4	0.6		

Table S1. *Cont.*

No.	Metabolite ID Matching									MS/MS Fragment Ion	Proposed Structure
	RT (min)	Formula $[M-H]^-$	Isotopic Peak No.	Calculated Mass	Observed Mass	Mass Diff. (ppm)	Calculated Abundance	Observed Abundance	Relative Error (%)		
**97**	24.3	$C_{25}H_{21}O_9$	1	465.1191	465.12	−2.01	100	100	0	–	Smiglabrone B or isomer
			2	466.1225	466.123	−1.02	27.6	26.7	0.9		
			3	467.125	467.1255	−0.96	5.5	5	0.5		
			4	468.1277	468.1252	5.26	0.8	0.7	0.2		
**98**	25.3	$C_{25}H_{21}O_9$	1	465.1191	465.1215	−5.25	100	100	0	–	Smiglabrone B or isomer
			2	466.1225	466.1244	−4.12	27.6	24.9	2.7		
			3	467.125	467.1272	−4.56	5.5	5.1	0.4		
			4	468.1277	468.1315	−8.08	0.8	0.6	0.2		
**99**	25.5	$C_{25}H_{21}O_9$	1	465.1191	465.1215	−5.2	100	100	0	335.0826, 341.0679, 323.0568, 231.0301, 217.0145	Smiglabrone B or isomer
			2	466.1225	466.1244	−4.03	27.6	24.7	2.9		
			3	467.125	467.1269	−3.94	5.5	4.3	1.2		
			4	468.1277	468.1291	−2.95	0.8	0.8	0		
**100**	25.9	$C_{25}H_{21}O_9$	1	465.1191	465.1208	−3.54	100	100	0	–	Smiglabrone B or isomer
			2	466.1225	466.1232	−1.55	27.6	27.2	0.4		
			3	467.125	467.1259	−1.75	5.5	4.8	0.7		
			4	468.1277	468.128	−0.69	0.8	1	0.2		
**101**	27.2	$C_{25}H_{21}O_9$	1	465.1191	465.1199	−1.73	100	100	0	–	Smiglabrone B or isomer
			2	466.1225	466.1223	0.48	27.6	33	5.4		
			3	467.125	467.1257	−1.37	5.5	7.2	1.7		
			4	468.1277	468.132	−9.16	0.8	1.3	0.5		

Table S1. *Cont.*

No.	Metabolite ID Matching									MS/MS Fragment Ion	Proposed Structure
	RT (min)	Formula $[M-H]^{-}$	Isotopic Peak No.	Calculated Mass	Observed Mass	Mass Diff. (ppm)	Calculated Abundance	Observed Abundance	Relative Error (%)		
**102**	31.4	$C_{25}H_{21}O_9$	1	465.1191	465.1215	−5.24	100	100	0	−	Smiglabrone B or isomer
			2	466.1225	466.1247	−4.77	27.6	29.4	1.8		
			3	467.125	467.1267	−3.57	5.5	6	0.5		
			4	468.1277	468.1312	−7.5	0.8	0.6	0.2		
**103**	32	$C_{25}H_{21}O_9$	1	465.1191	465.1202	−2.44	100	100	0	−	Smiglabrone B or isomer
			2	466.1225	466.1231	−1.21	27.6	26.5	1.1		
			3	467.125	467.1271	−4.34	5.5	4.9	0.6		
			4	468.1277	468.1273	0.96	0.8	0.5	0.3		
**104**	38.5	$C_{25}H_{21}O_9$	1	465.1191	465.1196	−1.09	100	100	0	−	Smiglabrone B or isomer
			2	466.1225	466.1231	−1.26	27.6	30.7	2.9		
			3	467.125	467.1233	3.65	5.5	5.5	0		
			4	468.1277	468.1307	−6.34	0.8	1.5	0.7		

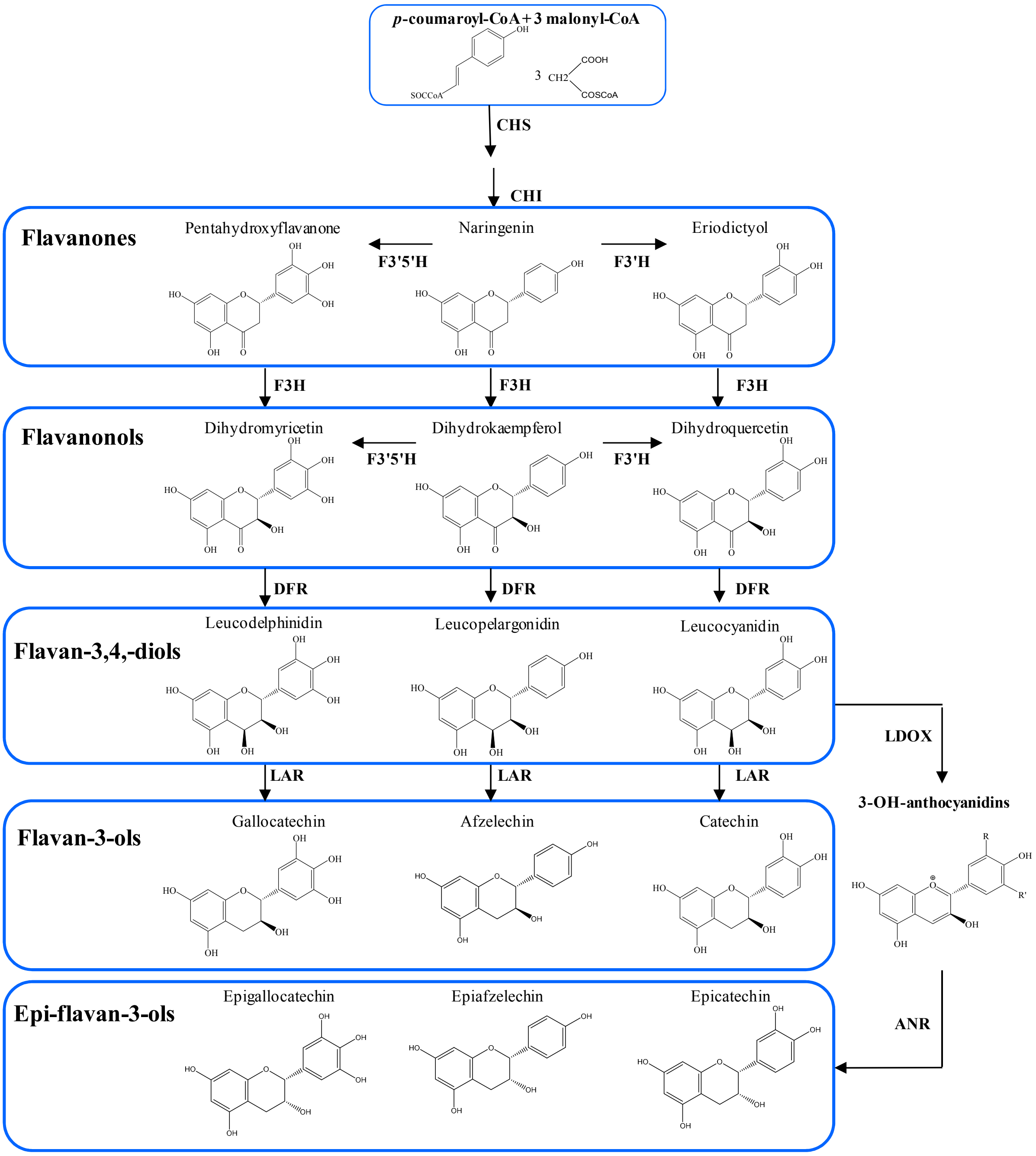

Figure S1. Flavonoid biosynthetic pathway (parts). CHS, chalcone synthase; CHI, chalcone isomerase; F3'5'H, flavonoid 3'5'-hydroxylase; F3'H, flavonoid 3'-hydroxylase; F3H, flavanone 3-hydroxylase; DFR, dihydroflavonol reductase; LAR, leucoanthocyanidin reductase; LDOX, leucoanthocyanidin dioxygenase; ANR, anthocyanidin reductase.
